# Supplementary material for: A Novel Splice Variant of the Inhibitor of Growth 3 Lacks the Plant Homeodomain and Regulates Epithelial–Mesenchymal Transition in Prostate Cancer Cells
Source: Biomolecules. 2021 Aug 4;11(8):1152. doi: 10.3390/biom11081152 (PMC8392754; doi:10.3390/biom11081152)
Supplement: Supplementary file 1 [file biomolecules-11-01152-s001.zip › biomolecules-1313910-supplementary.pdf]

A

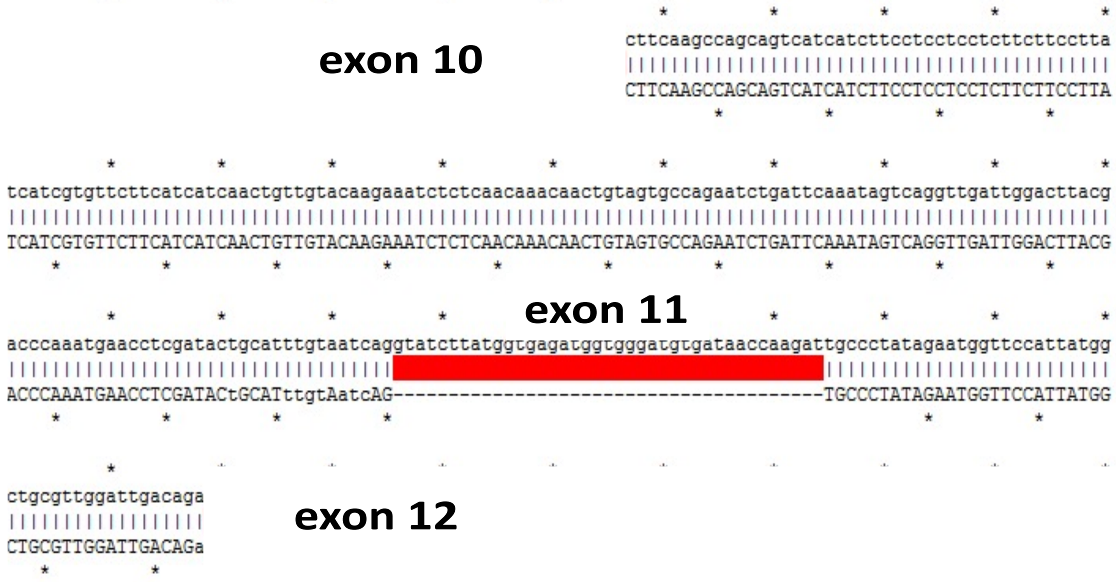

B

**ING1**	YCLCNQVSYGEMIGCDNDECPIEWFHFSCVGLNHKPKGKWYCPKC
**ING2**	YCLCNQVSYGEMIGCDNEQCPIEWFHFSCVSLTYKPKGKWYCPKC
**ING3 FL**	YCICNQVSYGEMVGCDNQDCPIEWFHYGCVGLTEAPKGKWYCPQC
**ING3Δex11**	YCICNQ-------------CPIEWFHYGCVGLTEAPKGKWYCPQC
**ING4**	YCLCHQVSYGEMIGCDNPDCSIEWFHFACVGLTTKPRGKWFCPRC
**ING5**	YCLCHQVSYGEMIGCDNPDCPIEWFHFACVDLTTKPKGKWFCPRC

Supplementary Figure S1. Sequence of in frame deletion of exon11 results in partial deletion of the PHD of ING3.

A) Sequence of exon 10-12 junction of full-length and ING3Δex11 splice variant. The deletion is highlighted in red.

B) Comparison of the PHD of ING1-5 with the ING3Δex11 splice variant indicating an in frame deletion within the PHD.

Fig. 1S

Supplement

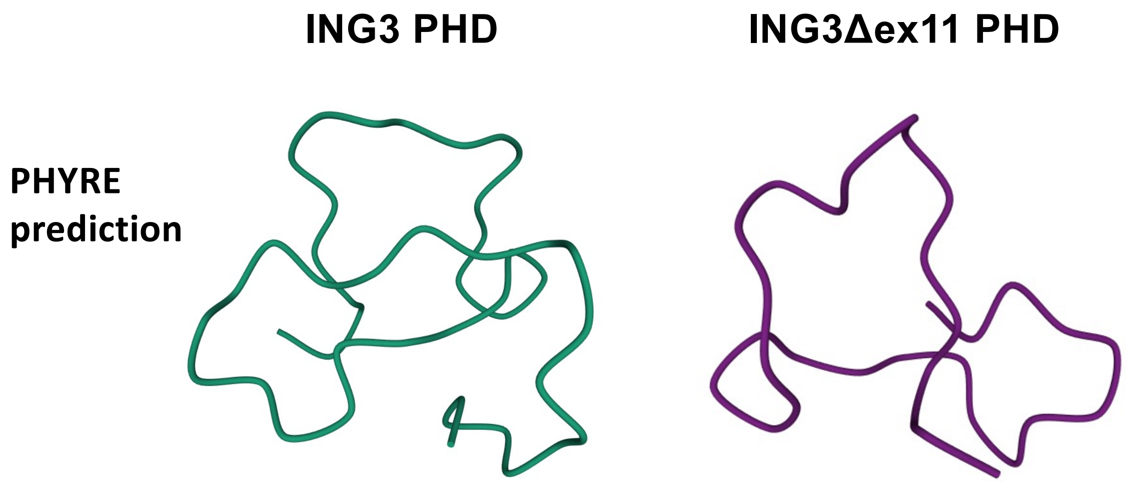

Supplementary Figure S2. Structure prediction using PHYRE software of the PHD of full-length ING3 and the remaining amino acids of the PHD sequence of the ING3Δex11 splice variant.

Fig. S2

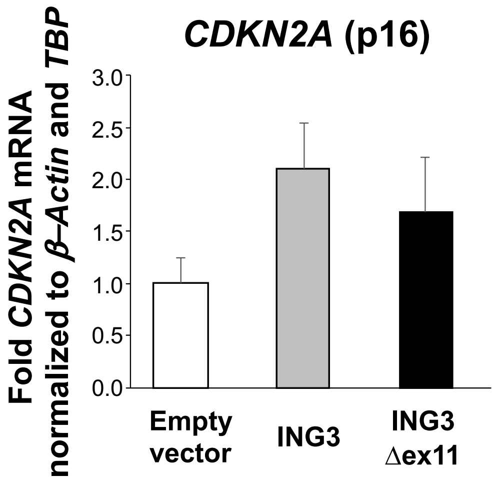

Supplementary Figure S3. *CDKN2A* exprerssion is induced by ING3 and ING3ΔEx11.
qRT-PCR from mRNA isolated from transfected LNCaP cells detecting the expression of *CDKN2B* encoding for the cell cycle inhibitor p16. Results of two independent experiments with each two replicates are shown. Values are normalized to the endogenous expression of the house-keeping genes β-Actin and TBP.

Fig. S3
